# The Role of 2-Oxoglutarate Dependent Dioxygenases in Gliomas and Glioblastomas: A Review of Epigenetic Reprogramming and Hypoxic Response

**DOI:** 10.3389/fonc.2021.619300

**Published:** 2021-03-25

**Authors:** Rebekah L. I. Crake, Eleanor R. Burgess, Janice A. Royds, Elisabeth Phillips, Margreet C. M. Vissers, Gabi U. Dachs

**Affiliations:** ^1^ Mackenzie Cancer Research Group, Department of Pathology and Biomedical Science, University of Otago Christchurch, Christchurch, New Zealand; ^2^ Department of Pathology, University of Otago, Dunedin, New Zealand; ^3^ Centre for Free Radical Research, Department of Pathology and Biomedical Science, University of Otago Christchurch, Christchurch, New Zealand

**Keywords:** hypoxia, brain cancer, PHD, HIF-1, TET, IDH, ascorbate

## Abstract

Gliomas are a heterogeneous group of cancers that predominantly arise from glial cells in the brain, but may also arise from neural stem cells, encompassing low-grade glioma and high-grade glioblastoma. Whereas better diagnosis and new treatments have improved patient survival for many cancers, glioblastomas remain challenging with a highly unfavorable prognosis. This review discusses a super-family of enzymes, the 2-oxoglutarate dependent dioxygenase enzymes (2-OGDD) that control numerous processes including epigenetic modifications and oxygen sensing, and considers their many roles in the pathology of gliomas. We specifically describe in more detail the DNA and histone demethylases, and the hypoxia-inducible factor hydroxylases in the context of glioma, and discuss the substrate and cofactor requirements of the 2-OGDD enzymes. Better understanding of how these enzymes contribute to gliomas could lead to the development of new treatment strategies.

## Introduction

**Figure 1:**
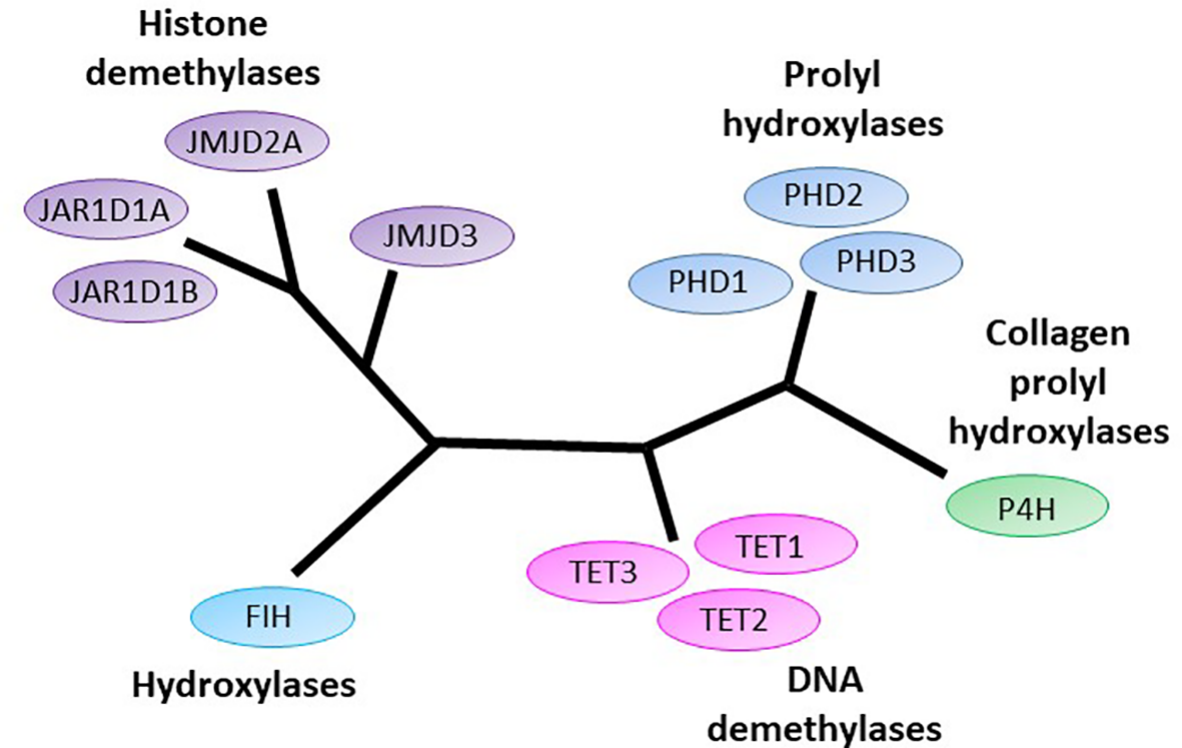
The human 2-oxoglutarate dependent dioxygenases implicated in initiation and progression of gliomas. 2-oxoglutarate dependent dioxygenases (2-OGDDs) include the DNA demethylases Ten-eleven translocases (TETs), the Jumonji-C domain containing histone demethylases (JMJD and JAR1Ds), the prolyl hydroxylases (PHDs) and hydroxylases that control hypoxic response (factor inhibiting HIF, FIH), and the collagen prolyl hydroxylases (P4H). Colors are used to indicate close phylogenetic relationships [adapted from Johansson (1)].

Gliomas and glioblastomas are brain cancers with significant morbidity and mortality, and limited treatment options. Our review will briefly describe these neoplasms, then concentrate on a super-family of enzymes, the 2-oxoglutarate dependent dioxygenase enzymes (2-OGDD), with dozens of members currently known (**Figure 1**). 2-OGDDs participate in numerous processes including collagen and hormone synthesis, fatty acid metabolism, stress signaling, epigenetic modifications and oxygen sensing (2–7). We will discuss specific members of the 2-OGDD family that have attracted recent interest, including the DNA demethylases [ten-eleven translocases (TET)], the histone demethylases [Jumonji-C domain-containing demethylases (JmjC)], and the hypoxia-inducible factor (HIF) hydroxylases. These enzymes require molecular oxygen and 2-oxoglutarate [2-OG, produced by isocitrate dehydrogenase (IDH)] as substrates, and non-ferrous iron (Fe^2+^) and vitamin C (ascorbate) as cofactors. Decreased availability of either substrate or co-factors reduces 2-OGDD enzyme activity, resulting in these enzymes acting as cellular sensors of energy metabolism, oxygen availability, and iron homeostasis (2–4, 8–14). Finally, we consider the potential means of modifying the activity of the 2-OGDDs, specifically by modulating ascorbate availability.

## Glioma and Glioblastoma and Treatment Options

Gliomas are a heterogeneous group of neoplasms that arise from glial cells in the brain (15, 16), or neural stem cells (17). Low-grade gliomas, grades II and III, include astrocytomas and oligodendrogliomas which predominantly develop from astrocytes or oligodendrocytes, respectively. Glioblastomas (GBM) are grade IV and can develop either as a high-grade lesion (primary GBM), or from astrocytoma progression (secondary GBM) (15, 16).

Gliomas accounted for 1.6% of cancer diagnoses and 2.5% of cancer related deaths worldwide in 2018 (18). Astrocytomas, oligodendrogliomas and secondary GBM tend to develop in younger individuals (median age 35, 45, and 38 years, respectively), compared to primary GBM (median age of 55 years) (17, 19). Gliomas are identified by magnetic resonance imaging (MRI) or computed tomography (CT/CAT). Although treatment options are available as detailed below, high grade gliomas are incurable (20–23). Some low grade gliomas can be cured, although these are rare (17). Following diagnosis, the median survival for patients with oligodendroglioma can be up to 16 years, for astrocytoma 5–8 years, and for GBM only 15–31 months (17, 24).

The current standard treatment for gliomas focuses on extending patient survival and includes maximal safe debulking surgery followed by radiotherapy and concomitant or adjuvant chemotherapy (20), usually with the alkylating agent temozolomide (20, 21, 25, 26). Debulking surgery is performed to reduce intracranial pressure and neurological symptoms, while resected tissue is utilized for tumor classification (17, 22, 23). Post-treatment recurrence is common with approximately 80% of gliomas recurring in close proximity to the primary site (27). Gliomas treated with temozolomide often hypermutate at recurrence leading to treatment resistance, as evidenced by the mere 20% of recurring gliomas showing response to the same agent (17, 28). Dissemination beyond the brain is uncommon, but some high grade gliomas may spread into the meninges or opposing brain hemisphere (17).

Besides traditional oncogenic drivers, gliomas are characterized by deregulated epigenetics and high levels of hypoxia (low oxygen), processes which are largely regulated by 2-OGDDs.

## 2-OGDD’s and Epigenetic Reprogramming

**Figure 2:**
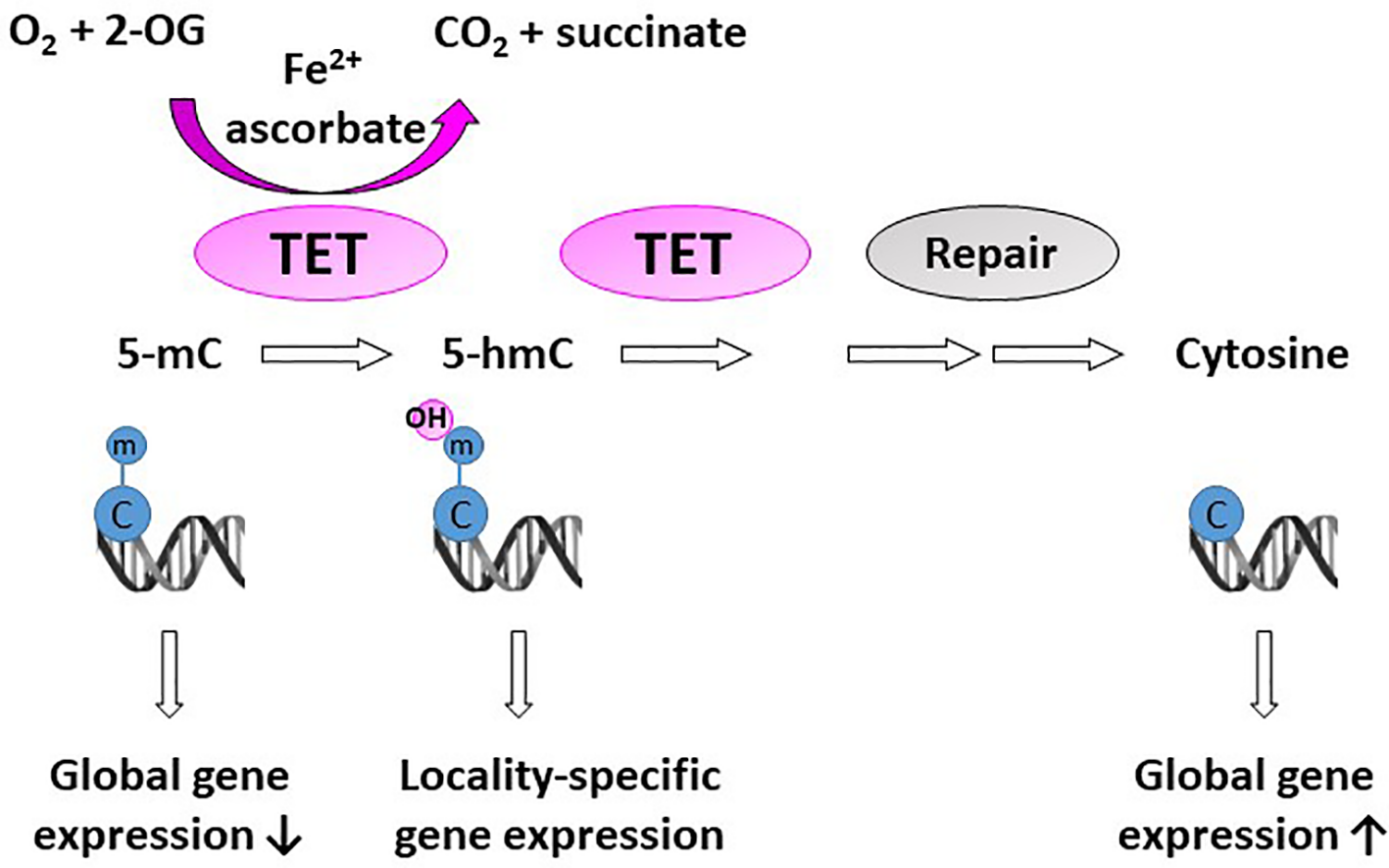
Activity of DNA demethylases. The ten-eleven translocases (TET1-3) hydroxylate methyl cytosine (5-mC). Hydroxy-methyl cytosine (5-hmC) are further converted to several intermediates, which are excised and repaired by thymine DNA glycosylase and base-excision repair (Repair) to generate an unmethylated cytosine. Methylation at CpG islands tends to repress gene expression, but some evidence suggests that gene expression is increased when 5-mC is converted to 5-hmC in gene promoters and enhancers (locality-specific); however, the exact biological role of 5-hmC is not yet known.

**Figure 3:**
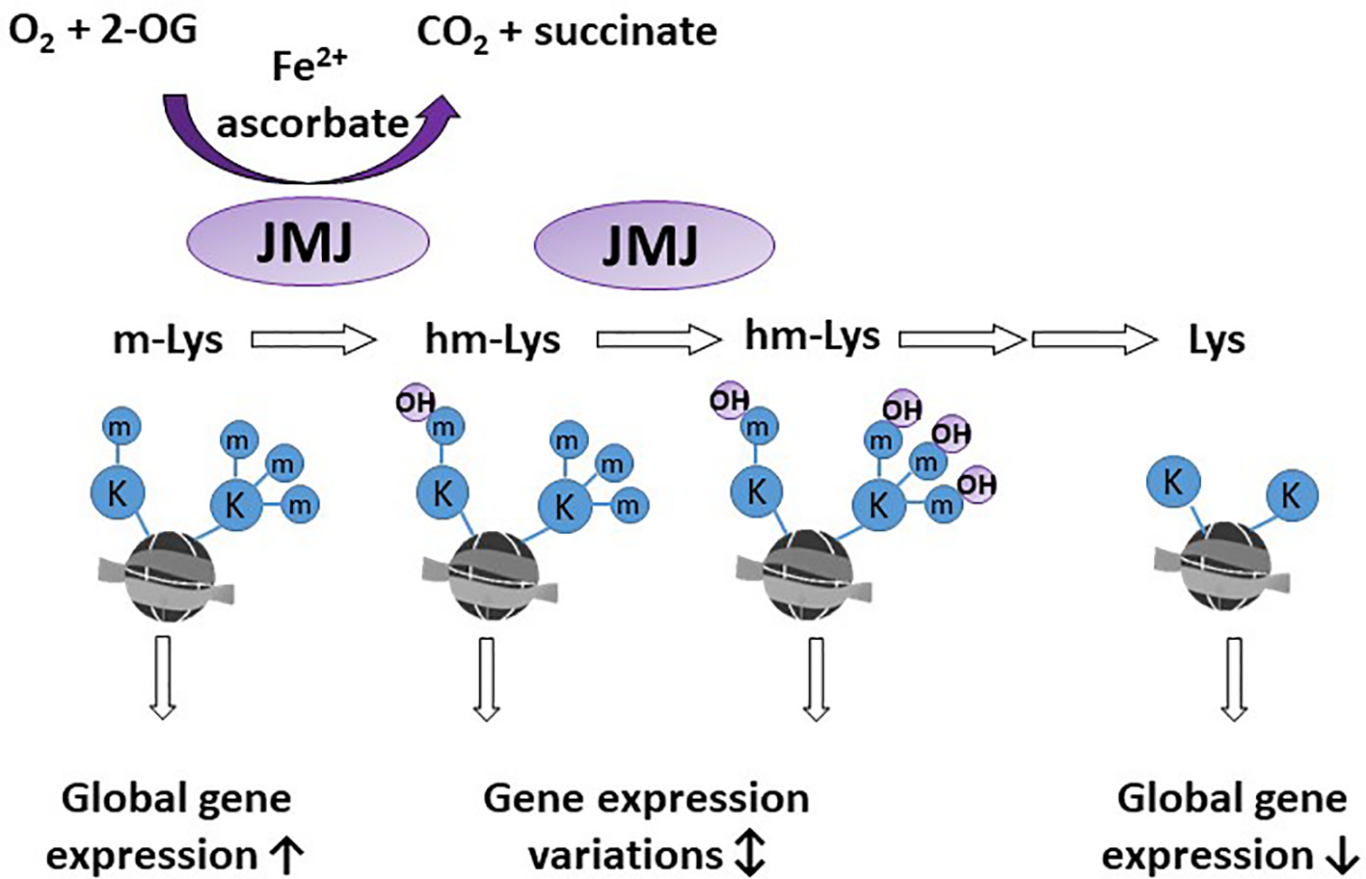
Activity of histone demethylases. Jumonji-C domain-containing demethylases (JMJ) act on lysine residues of the histone tails. Methylated lysine (m-Lys, mK) are hydroxylated (hm-Lys, hmK), which *via* several intermediates, leads to demethylated lysine residues. The effect of m-Lys and hm-Lys on histone structure and subsequent gene expression is very complex. Global gene expression tends to be increased in the more open methylated histone structure, compared to compact, demethylated histones.

A significant proportion of 2-OGDDs in mammals are demethylases involved in epigenetic reprogramming (6, 29). Methylation and demethylation of DNA and histones are the fundamental processes guiding epigenetic inheritance and regulating transcriptional activation and repression (30, 31). Consequently, aberrant modifications to these epigenetic processes, causing hyper- and hypo-methylating events, have emerged as hallmarks of cancer progression (32, 33). Demethylases regulate the epigenome through either the conversion of methylated cytosine (5-mC) to hydroxymethylcytosine (5-hmC) on DNA (**Figure 2**), or the removal of methyl groups from lysine residues on histones (**Figure 3**).

### Global Methylcytosine Status in Glioma

Epigenetic modifications are considered key mechanisms regulating occurrence and prognosis of glioma (34), and are used as classifiers of glioma subtypes (15, 16). Early genome-wide 5-mC analyses revealed that many low-grade gliomas and secondary GBMs contained large numbers of hypermethylated loci referred to as the glioma CpG island methylator phenotype (G-CIMP), and this was closely associated with the presence of somatic *IDH1* mutations, and improved prognosis (35–37). *IDH1* mutations are considered to occur early during the genesis of glioma, persisting during progression to secondary GBM, but they rarely appear in primary GBM (24, 38). Although initial reports suggested that G-CIMP remains stable during disease progression (35, 37), more recent analyses have shown their loss upon recurrence of *IDH1*-mutant gliomas (39–42). Loss of G-CIMP at recurrence resembled genome-wide 5-mC patterns seen in *IDH1* wild-type primary GBM, and was associated with poorer outcomes (41). A novel 7-CpG signature has been identified in non-G-CIMP primary GBMs, where high-risk signatures correlated with poorer overall survival in patients treated with temozolomide and radiation (43), suggesting that even in the absence of G-CIMP and *IDH1* mutations, 5-mC marks may be prognostic.

Interestingly however, earlier approaches for quantifying methylation, such as those used in the identification of G-CIMP (35, 36), relied on bisulfite conversion techniques, which cannot differentiate between 5-mC and 5-hmC. It was the introduction of oxidative bisulfide chemistry that made the distinction between 5-mC and 5-hmC possible (44), and consequently, led to the discovery of 5-hmC-specific binding proteins that are not only involved in DNA repair, but also transcriptional regulation (45, 46). These findings suggest that 5-hmC, in addition to being an intermediate in DNA demethylation, may have its own unique epigenetic role.

Many studies report a loss of global 5-hmC content in glioma compared to healthy brain tissues (47–52). Clinically, lower levels of 5-hmC have been associated with high tumor grade and poorer prognosis in glioma (48, 50, 53). More recent investigations moved beyond measuring global 5-hmC levels, to delineate, with base resolution, specific genomic locations to show 5-hmC patterns; all reporting higher than expected levels of 5-hmC at intronic CpG dinucleotides of high-grade gliomas and GBMs (50, 51, 54). Higher intronic 5-hmC levels correlated with elevated expression of the corresponding gene (54), with 5-hmC levels enriched within enhancer elements (50, 54), and 5-hmC levels associated with histone marks for open chromatin (50, 51). Thus, despite global loss of 5-hmC, genomic locations associated with transcriptional regulation and expression were commonly enriched for 5-hmC in glioma.

### Ten-Eleven Translocases (TETs)

The TET family consists of three members (TET1/2/3). TET enzymes are involved in both passive and active DNA demethylation (55). During active DNA demethylation, oxidized cytosine intermediates (5-hmC) are excised by thymine DNA glycosylase and repaired with base-excision repair (BER) to generate an unmethylated cytosine (56, 57) (**Figure 2**). Passive DNA demethylation on the other hand occurs during cell replication, when 5-mC at CpG sites are not recognized and replaced with unmethylated cytosine (58). In humans, TET1 is located on chromosome 10q21.3, TET2 on chromosome 4q24, and TET3 on chromosome 2p13.1. It is noteworthy that 10q21.3 is commonly deleted in gliomas (59–61).

TET2 is the most studied isoform in glioma. In comparison to normal human brain tissue, TET2 gene and protein expression is reduced in GBM and other gliomas (62, 63). TET2 expression is significantly decreased with increased grade, and lower TET2 was associated with poorer overall survival (62). Similar observations have been reported for the other two isoforms (53). In glioma, reduction in TET3 expression was associated with a genome-wide reduction in 5-hmC levels compared to normal brain, and decreased TET3 expression correlated with poorer prognosis (64). Together, these findings imply a tumor suppressive role for TET enzymes in glioma.

Numerous studies highlight the potential mechanisms by which TET expression and activity are dysregulated in gliomas. A likely mechanism for reduced TET activity is the indirect inhibition *via* mutated IDH1/2 (65). Mutant IDH1 enzymes often exhibit neomorphic activity, converting isocitrate into 2-hydroxyglutarate (2-HG), instead of 2-OG, which is required for TET activity (66, 67). In *IDH1*-mutant gliomas it has been suggested that 2-HG generation is responsible for the presence of G-CIMP, likely due to reduced TET demethylase activity (35, 36). Independent of IDH status, a complete absence of 5-hmC immunoreactivity was associated with nuclear exclusion of TET1 in 61% of gliomas (52). Transcription of TET2 may be repressed by zinc finger E-box-binding homeobox 1 (ZEB1) in gliomas. ZEB1 levels were inversely correlated with TET2 levels in tumors, and physical binding of ZEB1 to the TET2 promoter in glioma cells was observed *in vitro* (62). However, reductions in TET2 expression and activity are unlikely to be due to TET2 mutations, as direct sequencing of TET2 revealed very few mutations and minimal association of mutations with levels of 5-hmC in gliomas (68, 69). In humans, intragenic CpG sites within *TET2* showed higher levels of 5-mC and lower levels of 5-hmC in GBM compared to normal brain, although within the *TET2* promoter low levels of both 5-mC and 5-hmC were detected at CpG sites of normal brain and GBM (63). Therefore, it is unlikely that lower TET2 expression in glioma is transpiring as a result of promoter methylation-mediated transcriptional suppression, but may be an effect of transcription prevention due to intragenic DNA methylation. Interestingly though, in a cohort of low-grade gliomas that had promoter hypermethylation, all were wild-type *IDH1/2* (68). From this we hypothesize that in the absence of *IDH1/2* mutations, a small portion of IDH wildtype tumors may instead remodel TET2 promoter methylation in an attempt to disrupt TET2 activity. Taken together, these studies demonstrate the tumor suppressive role of TET enzymes in glioma, and highlight the potential mechanisms by which TET expression and activity may be dysregulated. Restoration of TET function to resemble healthy brain tissue may aid in regulating epigenetic processors that can counteract glioma progression and improve treatment outcomes.

### Jumonji-C Domain Containing Histone Demethylases

Many 2-OGDDs are from the large family of evolutionary conserved jumonji-C (JmjC) domain-containing proteins that are responsible for catalysing the removal of methyl groups from lysine residues on histones (29, 70) (**Figure 3**). There are several subfamilies of JmjC domain-containing histone lysine demethylases with substrate specificity to different methylated lysine residues, and this specificity is mediated by the functional domains unique to each subfamily (2, 29). The dynamic interplay between histone methylation and demethylation can result in either gene activation [via histone 3 lysine 4 (H3K4), H3K36 and H3K79] or inactivation (H3K9 and H3K27) (71, 72).

JmjC-domain containing demethylase isoforms are heterogeneously expressed in different brain locations and adult brain cell types (73, 74). Under hypoxic conditions, JMJD3 expression increased in neurons (75), but the modulation of specific histone methylation marks in response to varying oxygen tensions, and ranges of iron and 2-OG levels in brain cells, has yet to be determined.

In gliomas, changes in JmjC-dependent demethylase expression have been associated with differences in genome-wide histone methylation patterns (76, 77), and lower global histone methylation was associated with poorer patient survival (78). Conflicting reports on expression patterns of JmjC-dependent demethylases in glioma and GBM tissues have been published (77, 79–83). Overall, whether JmjC-dependent demethylases promote or suppress gliomagenesis is likely enzyme-dependent, although the majority of studies show these enzymes to promote tumor progression.

Introduction of the IDH1^R132H^ mutation in astrocytoma cells has been associated with both global histone hypermethylation (84), and enrichment of specific histone methylation marks (85). Mutant IDH1 enzymes generate the oncometabolite 2-HG instead of 2-OG, and 2-HG has been shown to competitively inhibit histone demethylase activity (65, 86). Despite the fact that reduced histone demethylase activity has been linked with higher histone methylation in astrocytoma cells, most clinical gliomas show increased levels of JmjC-dependent demethylases and lower histone methylation (76–81). Thus, further investigations of the biological mechanisms causing increased expression of JmjC-dependent demethylases in glioma is needed.

## 2-OGDDs and the Hypoxic Pathway

**Figure 4:**
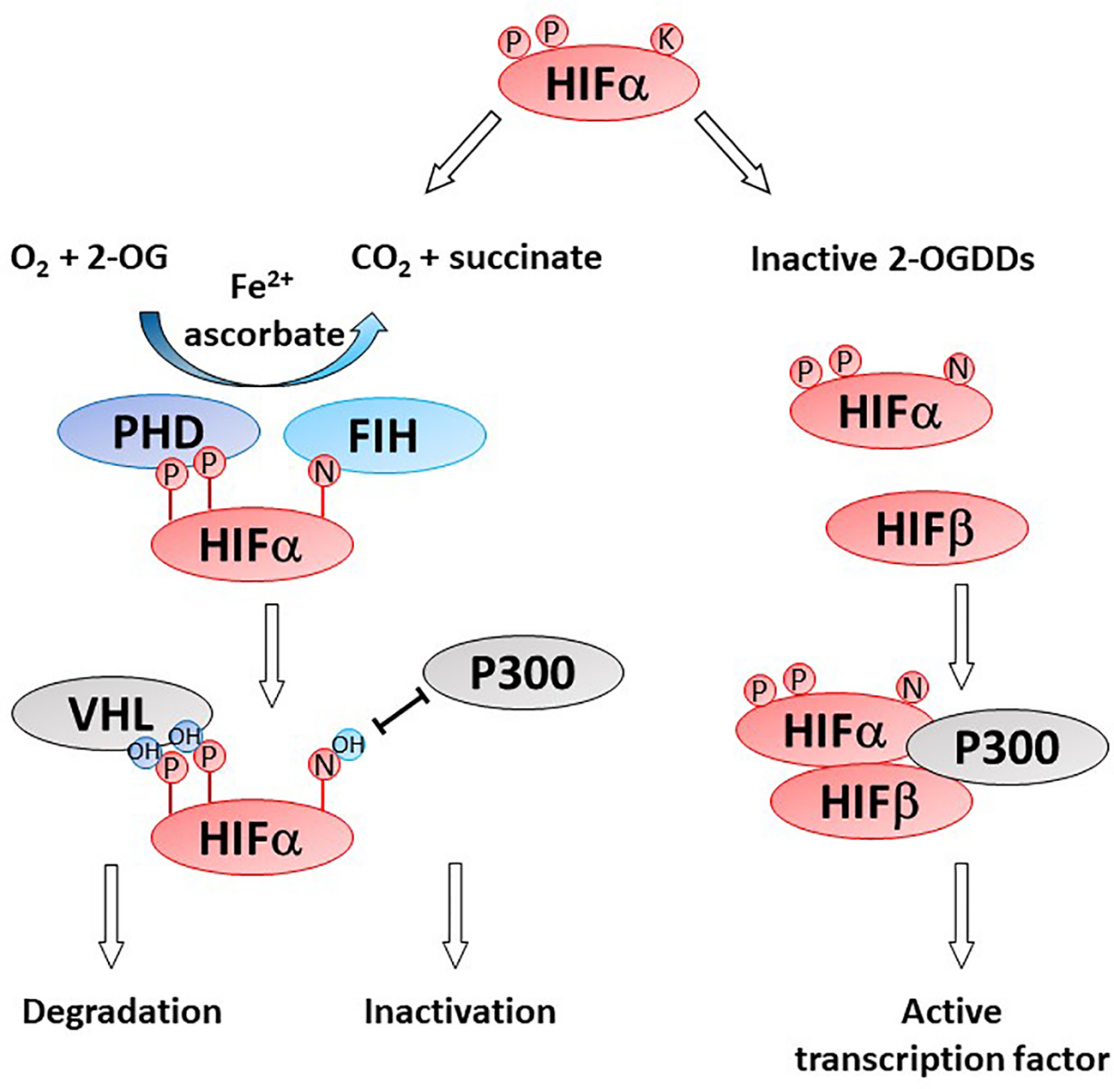
Activity of HIF-hydroxylases. Response to low oxygen (hypoxia) is regulated *via* the prolyl (PHD) and asparaginyl hydroxylases (factor inhibiting HIF, FIH). Specific proline (P) and asparagine (N) residues on the alpha subunit of the hypoxia inducible factor (HIFα) are hydroxylated by PHD and FIH, respectively. Proline hydroxylation enables recognition by von Hippel Lindau (VHL) as part of the proteasome and HIFα degradation. Asparagine hydroxylation prevent binding of the cofactor P300, thereby inactivating the HIF transcription factor. In the absence of one or more of the OGDD substrates or cofactors, PHD and FIH enzymes become inactive, enabling the accumulation of HIFα and formation of an active HIF transcription factor *via* binding to HIFβ. Genes under HIF control regulate cancer pathways such as angiogenesis, metastasis, glycolysis, etc.

Gliomas are highly hypoxic tumors and this is associated with poor patient prognosis (23, 87–89). Hypoxia induces adverse tumor characteristics including genomic instability, decreased apoptotic potential, increased expression of oncogenes and increased angiogenesis, which have all been described in gliomas (90). These characteristics are driven by the hypoxia inducible factors (HIFs) which are regulated by microenvironmental oxygen levels and the 2-OGDD enzymes, prolyl hydroxylases (PHD) and factor inhibiting HIF (FIH) (**Figure 4**) (91). As these HIF hydroxylases are dependent on oxygen for optimal function their activity is likely impaired in gliomas (92–95).

### Hypoxia-Inducible Factors

Hypoxic conditions typical of most solid tumors result in accumulation of the HIF transcription factors (11, 96). The HIFs (HIF-1,2,3) are heterodimeric transcription factors, consisting of one of three oxygen-sensitive α-subunits and a constitutive β-subunit (also known as aryl hydrocarbon receptor nuclear translocator (ARNT)) (97–99). HIF-1α is located on chromosome 14q23 (100); HIF-2α, also known as endothelial PAS domain protein 1 (EPAS1), is on 2p21, and HIF-3α is on 19q13 (101). Active HIF complexes accumulate in the nucleus and bind to specific hypoxic response elements (HREs) in promoter regions of HIF target genes, inducing their expression (102, 103). HREs contain the consensus sequence ^5’^CGTG^3’^, targets of CpG methylation, and CpG methylation blocks HIF-1 binding and transactivation (104). Even though HIF-1 and HIF-2 have identical HREs, their response to hypoxia, tissue distribution, target genes and their pro- or anti-tumor effects are distinct (105). Binding of HIF-1 and HIF-2 to their canonical HREs vary according to histone modifications, with HIF-1 preferentially associating with H3K4me3 modifications and HIF-2 with H3K4me1 (106). These binding patterns were interpreted as HIF-1 binding predominantly at regulatory regions within promoters, and HIF-2 binding to enhancer regions (106).

HIF’s modulate expression of hundreds of genes, and as a result, promote tumor growth and spread, adaptation to the tumor microenvironment, and resistance to chemo- and radio-therapy (107, 108). HIF activity also affects DNA methylation, histone acetylation and regulates noncoding RNAs (109), demonstrating the complexities of the hypoxic pathway.

HIF-1α has been proposed to drive glioma progression from low-grade astrocytoma to high-grade GBM (23, 110). Higher HIF-1α expression in human astrocytoma and GBM has been correlated with worse prognosis (111–114). Moreover, expression of HIF-1α, and its downstream target genes vascular endothelial growth factor (VEGF), glucose transporter (GLUT1), and carbonic anhydrase (CA9), show increased expression in higher grade gliomas compared to lower grade (115). In addition, increases in VEGF expression were shown to localize to hypercellular and necrotic regions that form as result of decreased oxygen and nutrient delivery (113, 116).

In comparison to HIF-1α, HIF-2α may be a specifically attractive target in GBMs, as it is expressed in glioma stem cells but not normal neuronal progenitors and it is activated by long-term hypoxia, in addition to its association with poor patient survival (117).

### HIF Hydroxylases

HIF activity is regulated at the post-translational level by two families of HIF hydroxylases, the prolyl hydroxylase domain (PHD) and factor inhibiting HIF (FIH) 2-OGDDs (**Figure 4**). PHDs hydroxylate specific proline residues on the HIF-α subunit targeting the subunit for proteasomal degradation (9, 92, 118). FIH hydroxylates an asparagine residue and prevents binding of the co-activators CBP/p300 and nuclear translocation (93, 119). The instability of HIF-α and the inhibition of transcriptional activators binding results in a reduction in both HIF-α protein and expression of target genes. Protein hydroxylation was considered irreversible, but a recent study showed evidence that FIH-mediated asparagine hydroxylation may be reversible by as yet unknown cellular enzymes (120).

The PHDs and the FIH enzymes have specific roles in oxygen and metabolic sensing due to their exquisite requirements for molecular oxygen and 2-OG, respectively (11). PHDs have higher affinity for oxygen (9, 13), bind unusually tightly to Fe^2+^ (121), and have a lower affinity for ascorbate compared to FIH (3), possibly due to the narrower opening to the enzyme active site of PHDs (122). PHD2 and FIH bind 2-OG with distinct residues, with FIH sharing more homology with JmjC-domain containing protein family of enzymes (94), suggesting evolutionary divergence (122). The HIF hydroxylases therefore have different sensitivities to loss of enzyme substrates and/or co-factors.

PHD2 (encoded by *EGLN1*) is generally acknowledged as the primary mediator of HIF-1α protein degradation, with PHD1 and 3 more likely to be involved in fine-tuning of the hypoxic response (123). This may be reflected in the subcellular localisation of each isoform, with PHD1 detected exclusively in the nucleus, PHD2 (and FIH) in the cytoplasm, and PHD3 in both compartments (124). Interestingly, PHD2 is activated, rather than inhibited, by the R-enantiomer of 2-HG as it mimics 2-OG, suggesting that IDH mutations may lead to HIF-α degradation (125–127). It has been shown, using immunohistochemistry, that HIF-1α and IDH^R132H^ expression are not related, supporting the ability of 2-HG to activate PHD2 (128).

*FIH* (also known as *HIF1AN*) is mapped to chromosome 10q24 (93). Full or partial deletions of chromosome 10q often occur in gliomas, and the frequency of these deletions increases with tumor grade (59–61). Thus, *FIH* mRNA expression often decreases with increasing tumor grade (129, 130). Investigation of FIH in GBMs has shown that FIH reduces the interaction between p300 and HIF-1α which is essential for transcriptional activity (131), and correspondingly higher FIH expression was associated with reduced *GLUT1* and *VEGF* expression in GBM cells (131). Loss of *FIH* through deletion of chromosome 10q24 may increase the hypoxic response, and thus contribute to an aggressive and treatment resistant glioma phenotype associated with hypoxia (23, 87, 88). While PHD2 is activated by the mutant IDH metabolite, 2-HG, FIH is inhibited, resulting in p300 interacting with HIF-α and inducing target gene expression (125). However, FIH has been shown to interact with a glioma tumor suppressor gene (*ANKDD1A*), which increased FIH activity, thereby reducing HIF-α activation and preventing HIF target gene expression (132).

## Substrate and Cofactor Requirements of 2-OGDDs

**Figure 5:**
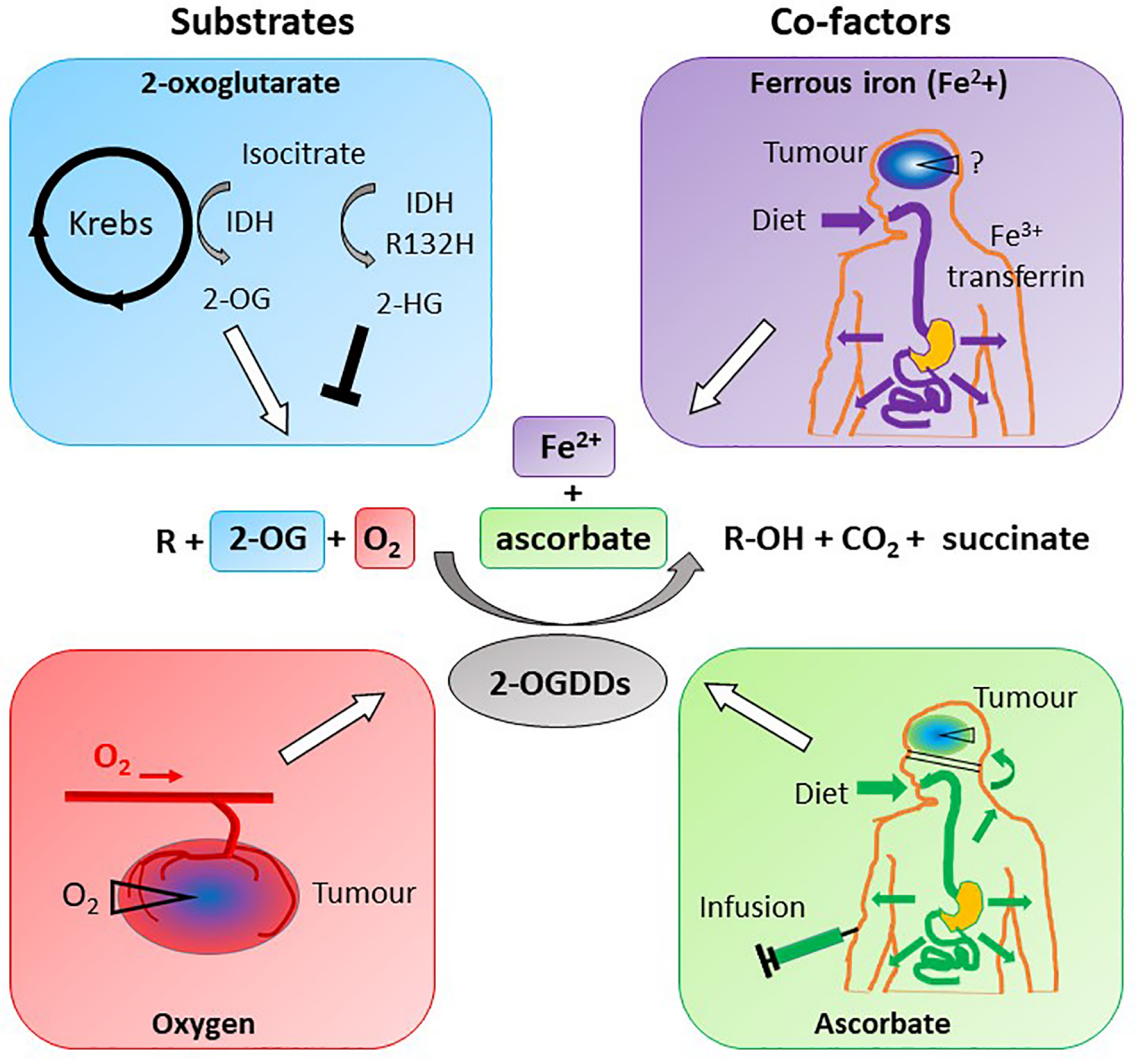
Substrate and cofactor requirements of the 2-oxoglutarate dependent dioxygenases. These enzymes hydroxylate nucleotides or amino acids (R), forming a hydroxylated product (R-OH). 2-OGDDs require 2-oxoglutarate (2-OG) and molecular oxygen (O_2_) as substrates, and ferrous iron (Fe^2+^) and ascorbate as cofactors. Isocitrate from the Krebs cycle is converted to 2-OG by isocitrate dehydrogenase (IDH), but mutant IDH (IDH^R132H^) produces the competitive inhibitor 2-hydroxyglutarate (2-HG). Low oxygen (hypoxia) is a characteristic of most solid tumors, thus reducing activity of 2-OGDDs. Iron obtained from the diet is transported *via* transferrin and taken up across the BBB *via* transferrin receptors; the content in tumors is unknown. Ascorbate is obtained *via* a healthy diet or infusions, and cannot cross the BBB but will travel *via* the choroid plexus. Cellular ascorbate uptake is *via* sodium vitamin C transporters; tumors may be low in the vitamin.

2-OGDDs contain a non-heme iron (Fe^2+^) in the active site, and utilize 2-OG (also known as α-ketoglutarate) and molecular oxygen as substrates (**Figure 5**). Ascorbate is an essential cofactor, and is thought to be required to maintain the active site Fe^2+^ in the reduced state (11–14, 133, 134).

### Molecular Oxygen as 2-OGDD Substrate and its Availability in Gliomas

Oxygen is an absolute requirement for all 2-OGDDs, but not all 2-OGDDs function as oxygen sensors. The PHDs have a K_m_ for oxygen of 230-250 μM, slightly above dissolved oxygen concentrations in air (20.9% O_2_ in gas phase ~ 200 μM dissolved O_2_) (135), and this property makes these proteins the predominant cellular oxygen sensors. These K_m_ values are significantly greater than for FIH at 90 μM, and the collagen prolyl 4-hydroxylases (C-P4H) at 40 μM (13, 135, 136).

Tissue partial pressure of oxygen (PO_2_) in the human body range from 104–108 mmHg in the alveola of the lung, to arterial PO_2_ of 90 mmHg and venous PO_2_ of 40 mmHg, to most tissues that have between tissue PO_2_ of 20–70 mmHg (137). Oxygen levels in the brain are heterogeneous and difficult to measure, with levels of 30–48 mmHg in normal brain recorded. Hypoxia is defined as <5 mmHg, and levels below 2.5 mmHg are known to induce clinical radioresistance (138–145). Theoretical modelling suggests the minimum oxygen level requirement for brain tissue is 17 mmHg and that a critical range for hypoxic injury is 4–11 mmHg (140).

Hypoxia is a prominent feature of gliomas. Oxygen levels in tumors have been measured using a range of techniques, including injection of hypoxia markers (146–149), direct measurements using polarographic O_2_ microelectrodes (145, 150) and MRI/CT scans (151). Using these techniques, oxygen levels were observed to be lower in gliomas and peritumoral regions compared to normal brain tissues, and readings below 2.5 mmHg were more common in tumors of increasing grade (145, 150, 152), with the most severe hypoxia detected in GBMs (23, 88, 89). A review of six studies reported a median PO_2_ of 13 mmHg in gliomas (144). This suggests that in high grade gliomas, the activity of 2-OGDD enzymes is likely to be reduced, but hypoxia is not routinely measured.

As ionizing radiation remains the mainstay of glioma treatment, and since hypoxia governs radioresistance, considerable clinical effort has been focused on reducing tumor hypoxia. With regards to 2-OGDD activity, these strategies may also improve enzyme activity, and are thus briefly discussed here. In an attempt to improve tumor oxygenation, patients have been given pure oxygen in a pressurized environment (hyperbaric oxygen) to breath (153). Alternatively, patients breathed 95% O_2_ with 5% CO_2_ (carbogen), which, together with hypercapnic-induced vasodilation, increases the amount of dissolved plasma O_2_ at the capillary level (154). Carbogen was tested in combination with nicotinamide, which is believed to prevent transient cessations in blood flow, thus inhibiting the development of acute hypoxia (154). Drugs have been developed to improve oxygen delivery (eg trans sodium crocetinate, TSC) (155), to normalize the tumor vasculature (e.g., anti-VEGF bevacizumab) (156), or to reduce oxygen consumption rate *via* mitochondrial poisons (e.g., anti-parasitic drugs atovaquone, ivermectin, proguanil, mefloquine, and quinacrine) and tested in patients with GBM (157). Overall, the results of these clinical trials have been disappointing and none of the approaches have been adopted into clinical practice, and actual oxygen measurements were largely lacking.

### 2-Oxoglutarate and Oncometabolites as 2-OGDD Substrates in Gliomas

The substrate 2-oxoglutarate (2-OG) is a product of the reaction in which isocitrate dehydrogenase enzymes (IDH) convert isocitrate to 2-OG (**Figure 5**). This reaction occurs in the TCA cycle *via* IDH3 and in the cytosol *via* IDH1 and IDH2. A number of *IDH1* and *IDH2* mutations have been reported in glioma, with the most common a base substitution in codon 132 of *IDH1* resulting in an arginine to histidine replacement (*IDH1* ^R132H^) (24, 67). IDH1^R132H^ has been identified in more than 70% of grade II/III astrocytic and oligodendroglial diffuse gliomas, and in more than 80% of secondary GBM, but rarely in primary GBM (24, 158, 159). IDH1^R132H^ enzymes generate 2-HG, instead of 2-OG (66, 67), which binds competitively to 2-OGDD enzymes and inhibits their function (**Figure 5**). Studies have, however, also reported that accumulation of 2-HG does not universally occur in all *IDH1* ^R132H^ and, conversely, that some wild type IDH cells accumulate 2-HG (160, 161). Further research is required to understand the impact of IDH mutations on patients with glioma, and how 2-HG accumulation interacts with other 2-OGDD substrates and co-factors to influence their activity in these tumors.

In gliomas with *IDH1^R132H^* mutations, drugs that inhibit the mutant IDH1 enzymes may improve 2-OGDD activity by reducing 2-HG production. A number of mutant IDH1 inhibitors are currently being evaluated in clinical trials with glioma patients, including AG-120 (Ivosidenib), AG-881 (Vorasidenib), BAY1436032 (Bayer), and DS-1001b (NCT02746081) (162–166). Pre-clinical investigations have reported anti-proliferative effects, reductions in tumor growth rates, and lower levels of 2-HG in both glioma cells and tumors from animal models (167–170). To date, 2-HG levels have only been measured in the plasma of human glioma patients following intervention with Ivosidenib, reporting no difference compared to those without treatment (163). Despite this, interest remains high and data from a Phase I study of Ivosidenib and Vorasidenib in patients with recurrent, non-enhancing, IDH1-mutant, low-grade glioma is currently pending (162). In addition to inhibitors, vaccines against mutant IDH1 have been tested in mice (171, 172). Mice with mutant *IDH1^R132H^* gliomas treated with the vaccine showed longer survival than non-immunized mice, and had higher levels of peripheral anti- IDH1^R132H^ antibodies, IFN-γ, and CD8+ T cells (172). However, 2-HG levels in tumors were not measured, and thus the vaccine effect on 2-OGDDs remains to be tested.

### Iron as Cofactor for 2-OGDDs and Availability in the Brain

Iron is the most abundant transition metal in the brain (173) and is more concentrated in some regions than in others (range; 13.5–1.75 µmol/g dry weight) (174), including the iron-rich substantia nigra, caudate nucleus and globus pallidus (175). Most gliomas arise in the frontal/temporal lobe, areas rich in glial cells and, potentially, relatively lower in iron. Astrocytes secrete hepcidin, which modulates the expression of ferroportin and other iron regulatory proteins, and thereby function as iron sensors to regulate and communicate the iron requirement of the brain through paracrine signaling (176–178).

Iron uptake into the brain is tightly regulated through the endothelial cells and neighboring astrocytes in the blood brain barrier (BBB) (176, 179, 180), primarily through the transferrin bound iron (TBI) pathway (174, 181, 182) however, when this pathway becomes saturated, non-TBI pathways are used (183). Through the canonical TBI uptake pathway, ferric iron (Fe^3+^) forms halotransferrin (174), which is able to pass through the BBB by binding to transferrin receptors on the apical surface of brain microvascular endothelial cells (BMVECs). Within the BMVEC, excess iron is stored in the cytosolic labile iron pool, which is the principle source of metals for metabolism (184). Efflux of iron through the abluminal membrane into the brain interstitium occurs through ferroportin (185, 186), expressed on the basolateral side of the BMVEC cells (186, 187). Once iron has crossed the BBB barrier, it is taken up by neurons, astrocytes, oligodendrocytes and microglia through TBI and nonTBI uptake pathways (183).

Iron plays a role in carcinogenesis, with proteins that modulate and regulate iron metabolism often dysregulated in gliomas (188–191). GBM cancer-stem-like cells were shown to upregulate transferrin expression, and to extract iron more effectively from the tumor microenviroment than non-stem-like tumor cells in an *ex vivo* explant model (192). The Fe^2+^ content in gliomas has not been reported but reduced levels may impede 2-OGDD function in tumors and cancer stem cells.

### Ascorbate as Cofactor for 2-OGDDs and its Availability in the Brain

As a cofactor for 2-OGDDs, ascorbate acts to reduce Fe^3+^ back to active Fe^2+^ (**Figure 5**). This activity appears to be specific to ascorbate as alternative reducing agents such as glutathione or N-Acetylcysteine are unable to substitute for ascorbate in 2-OGDD activity (12, 193, 194). Ascorbate is also involved in stabilizing cysteine residues in PHD enzymes, preventing intramolecular oxidation and supporting catalytic activity (126).

Normal brain tissue has one of the highest ascorbate levels of all tissues in the body, reaching intracellular concentrations of 2–10 mM depending on the cell type (195–197). In times of ascorbate insufficiency, the brain is one of the last tissues to lose ascorbate, supporting its importance to brain function (195, 198). The specific vitamin C transporters (sodium-dependent vitamin C transporters) are not expressed on the endothelial cells lining the BBB (198), and ascorbate enters the central nervous system through the choroid plexus where it can diffuse through the cerebrospinal fluid to the brain (195, 198). Cells within the brain express ascorbate transporters allowing intracellular ascorbate accumulation (198).

Data on ascorbate content in gliomas is limited to a single study that reported ascorbate levels in astrocytomas from eleven patients (199). While ascorbate levels were not different between astrocytoma tissue and non-neoplastic tissue, DNA content was significantly higher in astrocytoma (tumor) tissues indicating increased cell density (or cellularity) of the tissues. This suggests that intracellular ascorbate per cell may be reduced in astrocytoma tissue compared to normal, non-necrotic tissue (199). These intriguing findings need to be confirmed.

#### Ascorbate and Epigenetic Reprogramming

Evidence for the effects of ascorbate on epigenetic reprogramming is largely from embryonic cells (200, 201), but data in gliomas is missing. In patients with myeloid malignancies, oral ascorbate supplementation resulted in an increase in the ratio of 5-hmC compared to 5-mC in mononuclear myeloid cells (202). Here, DNA demethylation was not associated with changes in TET expression (202, 203), but instead appear to result from ascorbate-mediated restoration of endogenous TET activity, which was supported by studies with *Tet2-*deficient mice (204). Gliomas show lower TET expression independent of *TET* mutations (63, 68, 69), and we hypothesize that ascorbate may compensate for lower TET expression by upregulating residual TET2 function as was observed in a case study of acute myeloid leukemia (205).

Low-grade primary gliomas commonly harbor *IDH* mutations that persist during progression to secondary GBM (24). *In vitro*, ascorbate was able to circumventing competitive inhibition by 2-HG in colon cancer and HOXA9-immortalized mouse bone marrow cells with IDH1^R132H^ mutations (206, 207). One *in vitro* study reported the effects of ascorbate on epigenetic marks in LN229 glioma cells, showing increased *TET3* mRNA expression, as well as increased 5-hmC, but these cells did not harbor an IDH mutation (64). Yet, these findings, together with reported associations between higher 5-hmC levels and better prognosis in patients with GBM (50), support the notion that sufficient ascorbate levels in glioma tumors may induce demethylation activity and promote more favorable outcomes, although whether sufficient ascorbate can overcome high levels of 2-HG remains to be determined.

In addition to TET-mediated effects, ascorbate induced H3K9me2/3 and H3K36me2/3 demethylation *via* JmjC-dependent demethylases in embryonic stem, embryonic fibroblast and Th17 cells from mice (208–210). Ascorbate caused reductions in H3K9 methylation and increased expression of the JmjC-dependent demethylases, JHDM2A-C and JHDM3B, as well as widespread DNA demethylation at CpG island boundaries in human embryonic stem cells (211). However, investigations in gliomas are lacking, despite the well-established link between glioma formation and global 5-hmC deficiency.

#### Ascorbate and the Hypoxic Pathway

The relationship between intracellular ascorbate levels and HIF pathway activity has been investigated in numerous cancers, but not yet in gliomas. *In vitro* investigations of intracellular ascorbate levels and HIF pathway activity have been performed in varying cancer types, findings from which have guided further *in vivo* studies (212–215). In relevant mouse models (using ascorbate-dependent Gulo^-/-^ mice), increased ascorbate intake or administration was associated with increased tumor ascorbate levels, reduced HIF pathway activity and reduced tumor growth (216). In clinical samples of endometrial (217), colorectal (218), thyroid (214), papillary cell renal cell carcinomas (215, 219), and breast cancer (220), high tumor ascorbate levels were associated with low HIF-1α protein levels and low HIF target gene expression. This relationship was not evident in clear cell renal cell carcinoma (215, 219), that have a mutated von Hippel-Lindau factor which prevents proteasomal degradation of hydroxylated HIF-α (219). Higher levels of tumor ascorbate were associated with improved disease-free survival in patients with colorectal cancer (218) and improved disease-specific survival in patients with breast cancer (220).

## High Dose Ascorbate as Cancer Treatment

Infusion with high dose ascorbate as an alternative or complementary therapy for cancer is widespread (221), but lacks evidence of efficacy (222, 223), despite early promising data (224, 225). Since then, pharmacokinetic data have demonstrated that infusion results in supra-physiological plasma ascorbate levels that are not achievable by oral administration (226–229). Case studies and small clinical trials continue to surface that suggest there may be circumstances under which high dose ascorbate infusion can provide a clinical benefit (230–239).

### Preclinical Models of High Dose Ascorbate Treatment in Gliomas

In a mouse xenograft glioma model, analysis of ascorbate levels in plasma, tumor, and cerebrospinal fluid samples showed that ascorbate increased 1 h post intraperitoneal injection with 4 g/kg of ascorbate (240). One study, using an intracranial GL261 glioma mouse model, reported that radiation treatment slowed tumor growth, whereas ascorbate treatment made no difference, and the combination of ascorbate and radiotherapy induced faster progression (241), in conflict to *in vitro* findings of radio-sensitisation by ascorbate in numerous glioma cell lines, including GL261 cells (242–244). However, ascorbate levels in the intracranial model were not measured, and thus the impact of ascorbate on glioma response to radiation remain uncertain.

### Clinical Trials in Patients With Gliomas and GBMs

Preclinical data led to phase I clinical trials administering intravenous ascorbate to glioma patients, with and without standard radiotherapy and temozolomide (245). High dose vitamin C (HDVC) was found to be safe and well tolerated, reaching target 20 mM plasma levels (240, 245), but tumor ascorbate levels were not measured. A trend of improved overall survival was reported, but participant numbers were too small to determine statistical significance (240). Two case reports for the use of HDVC infusions in patients with glioma have also been reporting (237, 246).

Previous research has shown an association between a lower proportion of methylation at the O-6-methylguanine-DNA methyltransferase (MGMT) promoter in glioma tumors and poorer patient prognosis (247). MGMT is a DNA repair enzyme responsible for resistance to temozolomide, and hypermethylation of the *MGMT* promoter is evident in 40–45% of gliomas (248, 249), with higher methylation levels in low-grade gliomas compared to GBMs (53). Interestingly, in glioma patients with low methylation levels at the *MGMT* promoters, HDVC infusions resulted in improved overall survival (240), but unfortunately, ascorbate levels in the glioma tissue were not measured. Plasma ascorbate levels do not necessarily reflect tumor ascorbate levels due reduced functioning vasculature and BBB in gliomas. Overall, the clinical worth of HDVC in cancer remains unproven.

## Conclusion

The superfamily of 2-OGDD enzymes play a vital role in glioma progression and patient prognosis, being involved in epigenetic modifications and oxygen sensing. Limiting supplies of one or more of their substrates or cofactors in gliomas is likely although reported measurements are rare. Restoration of epigenetic modifications offers a promising target in the treatment of cancer, as these alterations are reversible, as opposed to genetic mutations. Attempts at increasing tumor oxygenation to improve effectiveness of radiation and chemotherapy in glioma are not (yet) in clinical practice (250), and new strategies are sought. Ascorbate infusion is a safe and cheap option that may be able to normalize 2-OGDD function in a subset of glioma tumor subtypes. However, this will likely depend on mutation status and on the ability to increase intracellular ascorbate levels in these tumors. Future research will need to confirm ascorbate status of clinical glioma tumors, on measuring 5-hmC levels and HIF activity in clinical samples, and on determining an optimal ascorbate dose for patients, before embarking on phase III trials to determine clinical efficiency.

## Author Contributions

Conceptualization, RC, EB, JR, and GD. Funding acquisition, EP and GD. Supervision, EP, MV, and GD. Writing—original draft, RC and EB. Writing—review and editing, JR, MV, and GD. All authors contributed to the article and approved the submitted version.

## Funding

We received funding from the Mackenzie Charitable Foundation (EP and GD), the Canterbury Medical Research Foundation (RC, EP, and GD) and the University of Otago (RC and GD).

## Conflict of Interest

The authors declare that the research was conducted in the absence of any commercial or financial relationships that could be construed as a potential conflict of interest.
